# *Curcuma longa* L. Rhizome Essential Oil from Extraction to Its Agri-Food Applications. A Review

**DOI:** 10.3390/plants10010044

**Published:** 2020-12-28

**Authors:** María Dolores Ibáñez, María Amparo Blázquez

**Affiliations:** Departament de Farmacologia, Facultat de Farmàcia, Universitat de València, Avd. Vicent Andrés Estellés s/n, 46100 Burjassot, València, Spain; mijai@alumni.uv.es

**Keywords:** *Curcuma longa*, essential oil, extraction methods, chemical composition, agri-food industry, antimicrobial, herbicidal, antioxidant

## Abstract

*Curcuma longa* L. rhizome essential oil is a valuable product in pharmaceutical industry due to its wide beneficial health effects. Novel applications in the agri-food industry where more sustainable extraction processes are required currently and safer substances are claimed for the consumer are being investigated. This review provides information regarding the conventional and recent extraction methods of *C. longa* rhizome oil, their characteristics and suitability to be applied at the industrial scale. In addition, variations in the chemical composition of *C. longa* rhizome and leaf essential oils regarding intrinsic and extrinsic factors and extraction methods are also analysed in order to select the most proper to obtain the most efficient activity. Finally, the potential applications of *C. longa* rhizome oil in the agri-food industry, such as antimicrobial, weedicide and a food preservative agent, are included. Regarding the data, *C. longa* rhizome essential oil may play a special role in the agri-food industry; however, further research to determine the application threshold so as not to damage crops or affect the organoleptic properties of food products, as well as efficient encapsulation techniques, are necessary for its implementation in global agriculture.

## 1. Introduction

Medicinal and aromatic plant species (MAPs) have been broadly exploited as food flavourings, medicinal agents, preservatives and ornaments, as well as beauty and personal delight products, becoming natural alternatives that offer reliability, safety and sustainability [1,2]. Amongst them, turmeric (*Curcuma longa* L., *Zingiberaceae*) is especially popular worldwide because of its attractive culinary, cosmetic and medicinal uses [3]. Specifically, the interest of this tuberous species resides in its exploitation as a colouring and flavouring agent, as well as in its numerous pharmacological activities, such as antioxidant, anticancer, anti-inflammatory, neuro- and dermoprotective, antiasthmatic or hypoglycaemic [4,5,6,7,8,9,10], being recently reported that turmeric can even potentially contribute against the life-threatening viral disease COVID-19 by inhibiting the main protease enzyme [11]. Most of these interesting features and properties principally come from the rhizome [3,12], a horizontal underground stem from which the shoots and roots arise [13]. It has distinctive organoleptic properties: a yellow/brown colour externally, with a deep orange inner part, a special aromatic smell and a bitter, hot taste. These characteristics make *C. longa* rhizome ideal for gastronomy. Especially, it is the principal ingredient of curry, for which it is probably popularly known [14,15,16].

Furthermore, rhizomes are a rich source of two major products with remarkable attributes: curcuminoids and essential oils [17]. On the one hand, curcuminoids are the responsible for the previously described orange-yellow colour [15]. They particularly refer to a group of three phenolic compounds, curcumin, desmethoxycurcumin and bisdesmethoxycurcumin, belonging to the diarylheptanoid family. They consist of a diketonic hydroxycarbon skeleton with different functional groups, depending on the curcuminoid [18,19]. Their content in the *C. longa* rhizome may vary according to many factors, such as the variety and geographic location, as well as cultivation and postharvest processing conditions [17,20]. For these secondary metabolites, turmeric is commonly employed as a spice and additives that provide colour and flavour in the food industry [21]. Additionally, they have demonstrated promising antioxidant and anti-inflammatory activities, being considered a valuable complementary therapy to pharmaceuticals in Crohn’s, diabetes and cancer between other disorders [15,21,22]. Unfortunately, their poor solubility, low absorption and bioavailability, as well as high metabolic rate, limit their use for therapeutic purposes [23,24,25,26]. In fact, the major component curcumin has not been approved as a therapeutic agent yet due to its pharmacokinetics and physicochemical properties, despite it is generally considered a safe substance [24,27]. In response, curcuminoids have been associated with lipids, micelles, nanoparticles and other molecules to enhance their effects. An example is the binding of curcumin with phosphocaseins. This combination represents a suitable vector to deliver efficiently the compound, as well as other drugs and nutrients in general. New analogues with improved activity are being tried to develop from the original ones [21,28,29,30,31,32].

On the other hand, the essential oil is the one that provides the *C. longa* rhizome a particular spicy and aromatic flavour [3,15] with its distinctive chemical composition. In general, sesquiterpenes are the predominant phytochemical group in *C. longa* rhizome oil [33]. More concretely, ar-, α- and β–turmerones are usually the major and most representative components [34,35], although numerous intrinsic and extrinsic elements may influence in their quality and quantity [36,37,38,39,40,41]. Nevertheless, this chemical composition is different from the essential oil extracted from the aerial parts in which monoterpenes (α-phellandrene, terpinolene, 1,8-cineole, etc.) stand out [42,43,44,45,46]. Countless beneficial health effects have been attributed to *C. longa* rhizome oil as a consequence of this particular chemical composition: cardiovascular protection, antihyperlipidemic, antiglycaemic, antioxidant, antiplatelet, anti-inflammatory, antioxidant, antiarthritic, etc. [47]. Especially, abundant research has been focused on ar-turmerone, demonstrating its promising interesting medicinal properties, like the protection against the development of certain tumours [48,49], antifungal activity against dermatophytes [50], antiangiogenic effects [51], anticonvulsant properties [52] and treatment of neurodegenerative and other inflammatory diseases, such as psoriasis [53,54].

Nowadays, there is a growing demand of essential oils in the perfume and cosmetics, agriculture, pharmacy, food and beverage, as well as in many other, industries. One of the principal aims is to replace synthetic products with detrimental health and environmental effects [55]. In particular, numerous essential oils such as winter savoury, peppermint, oregano, wintergreen and eucalypt, as well as many of their principal components (carvacrol, limonene, etc.) have already exhibited attractive and useful antimicrobial, herbicidal and antioxidant activities for the agri-food industry [56,57,58,59,60,61,62]. These data favour their potential use as natural preservatives to prevent the crop and food spoilage and extend the shelf-life, as well as weed control without significantly affecting the harvests.

The medicinal and culinary properties of *C. longa* rhizome oil are well-known. However, its potential applications in the agri-food industry are still under investigation. Therefore, the attempt of the present review is to present detailed literature dealing with the extraction, chemical composition and biological activity of *C. longa* rhizome essential oil in order to highlight the potential application in the agri-food industry as natural, safer and more sustainable antimicrobial, herbicidal and antioxidant agents. Specifically, the different possible extraction methods of the essential oil from the rhizomes *C. longa* and their characteristics will be discussed first. Then, the qualitative and quantitative chemical compositions of *C. longa* rhizome oil and the factors that influence it, as well as the difference with other parts of the plant and other *Curcuma* spp. will be described. Finally, the antimicrobial, herbicidal and food preservative properties of *C. longa* rhizome oil will also be discussed to assess a prospective application in the emergent “bio” agri-food industry.

## 2. Extraction Methods to Obtain Essential Oil from *C. longa* Rhizomes

The characteristic aroma of turmeric’s rhizomes is provided mainly by its essential oil, representing an excellent marker of quality of this spice and its derived products. Several extraction processes have been carried out with the subterranean plant stems to obtain this mixture of flavouring compounds, steam distillation being the most commonly chosen one [63,64,65]. In this process, a blast of steam goes through the plant material placed on a perforated plate above, dragging the organic compounds [66,67]. It presents certain disadvantages on an industrial scale (Table 1), including the huge amounts of raw material and time required and, consequently, the high price [68]. In addition, this process can present difficulties sometimes, either the evaporation of the steam-volatile compounds by the remaining latent heat or the collapse by their excessive elevation in the flask [69,70]. With the aim of avoiding these drawbacks and consequently increasing the quality and quantity of the essential oil, this technique has usually been modified and/or combined with others. For instance, Chandra et al. incorporated a continuous water circulation process to the regular steam distillation of the essential oil of turmeric rhizomes and leaves, achieving 13% and 29% more yield, respectively, compared to the conventional process [71]. Moreover, a subsequent distillation with vacuum allows a more efficient extraction of turmeric monoterpenes and sesquiterpenes [72]. The addition of a packed bed of turmeric rhizomes above the steam source has been also key to maximize the essential oil yield [73]. In general, a steam jacket is formed, helping reach a constant elevated temperature of the distillation and avoiding the degradation of the oil and, therefore, the unwanted odours that emerge from it [73,74] (Table 1).

On the other hand, hydrodistillation is also widely employed in the extraction of the essential oils from turmeric rhizomes on an industrial scale, due to its low-cost efficiency and easy implementation [75]. Unfortunately, it may sometimes mean longer extraction times and the production of wastewater, as well as loss and alteration in the composition of essential oils because the raw material is in contact with the boiling water [74,75]. Despite this, distillation in the Clevenger apparatus gives better results in the deodorisation process of turmeric relative to other distillation methods, such as distillation using the Kjeldahl apparatus or under high vacuum [76].

The most recent extraction methods appear to overcome the limitations of the conventional ones, such as heat transfer, time and quality of the resulting essential oil [77,78]. These advantages have also been observed in the extraction of oleoresins and, more particularly, curcuminoids, active components of the dried rhizome of *C. longa* extracts [79,80,81].

Amongst these methods, supercritical fluid extraction (SFE) [65,70,82] has shown many advantages for the extraction of essential oils on an industrial scale, including the reduction of extraction times, higher quality extracts and, principally, the use of carbon dioxide (CO_2_) as a nontoxic, non-flammable and free-of-residues solvent [74,83,84,85]. In relation to turmeric, superior yields but no significant differences in the relative composition or higher concentrations of most of the essential oil components [79] were obtained using SFE rather than the conventional systems of steam distillation and ultrasound extraction. However, the turmeric oil yield was higher with Soxhlet extraction than SFE [86,87]. Particularly, the combination of 320 K and 26 MPa gives an optimum production of turmeric oil with 71% turmerones’ purity [88] or 67.7% with 313 K and 20.8 MPa [89]. Similar optimal conditions to obtain the highest-quality essential oils from turmeric rhizomes (75% of ar-, α- and β-turmerone) were reported by Carvalho et al. (333 K and 25 MPa) [90]. Nevertheless, this technique is still under study to achieve a higher optimization. The influence of the variation of different operating parameters (temperature, extraction time, pressure, solubility and particle size) together with the integration of other techniques, such as SFE assisted by pressing (SFEAP), are investigated to reach higher yields, the quality of turmeric essential oil and its main compounds [82,83,91,92] (Table 1).

Among SFE, subcritical water extraction (SWE) also demonstrated many advantages over traditional methods in the recovery of bioactive compounds from plants, excepting implementation on the industrial scale for the moment [93]. Specifically, it takes advantage of the special properties of supercritical water under high temperature and pressure conditions (100–374 °C, >50 bar) to extract nonpolar compounds [94]. After a deep study of the influence of operating conditions in the extraction of *C. longa* essential oil from rhizomes (temperature, flow rate, particle size, time, etc.), SWE has demonstrated its selectivity to enhance a target compound and its suitability as a green and effective method for the extraction of essential oil and curcumin from turmeric rhizome [86] (Table 1).

**Table 1:** Different extraction methods to obtain *Curcuma longa* essential oil: advantages and limitations. SFME: solvent-free microwave extraction, MAE: microwave-assisted extraction, HDAM: hydrodistillation assisted by microwave, SDAM: steam distillation assisted by microwave, VMHD: vacuum microwave hydrodistillation and MHG: microwave by hydrodiffusion and gravity. ↑: Increase, ↓: Decrease.

**Extraction method**	**Steam Distillation**	Advantages	Can be modified and/or combined with other techniques to maximize the yield and efficiency, e.g., ↑13–29% yield	[63,64,65,71,74]
		Limitations	Huge amounts of raw material needed Time-consuming High price Evaporation of steam-volatile compounds and even collapse	[68,69,70]
	**Hydrodistillation**	Advantages	Low-cost efficiency Easy implementation Clevenger gives better deodorization results than other processes	[75,76]
		Limitations	Long extraction times Production of wastewater Loss and/or alteration in the composition of essential oils	[74,75]
	**Supercritical Fluid Extraction**	Advantages	Reduction of extraction times Higher quality extracts CO_2_ as nontoxic, non-flammable and free-of-residues solvent Superior yields	[73,79,83,84,85]
		Limitations	No significant differences in qualitative and quantitative composition of turmeric essential oil with respect to other methods: 67.7–75% turmerone purity at 313–320 K and 20.8–26 MPa Under study to achieve higher optimisation	[79,82,83,88,89,91,92]
	**Subcritical Water Extraction**	Advantages	Especially useful to extract non-polar compounds Selective to enhance a target compound Green and effective to extract the essential oil and curcumin	[86,94]
		Limitations	Low implementation in industry currently	[93]
	**Ultrasonic Extraction**	Advantages	Improved mass transfer between plant cell and solvent Combination with other techniques: ↑ efficiency, ↓ processing time, ↓costs	[68,82,95]
		Limitations		
	**Microwave Energy (SFME, MAE)**	Advantages	↓ Costs ↓ Extraction times ↓ Energy consumption ↓ CO_2_ emissions Combination with other techniques to improve the performance: HDAM, SDAM, VMHD, MHG ↓ Extraction time from 4 h of hydrodistillation to 1 h No degradation products Maximum yield	[96,97,98]
		Limitations		
	**Solvent Extraction**	Advantages	Overcomes the problem of excessive heat; avoids the loss of compounds and properties of the essential oil Suitable and safe extractants: chloroform and freons	[99,100,101]
		Limitations		

Ultrasonic extraction is another method of extraction of essential oils and other bioactive compounds [77,95]. It is based on ultrasonic cavitation: a bubble implosion produces micro-jets that destroy the lipid glands in the plant cell tissue, releasing the essential oil [68]. It has overcome low extraction kinetics and yields of SFE-resulting essential oils by enhancing the mass transfer between the plant cell and solvent [68,95]. Moreover, it is usually combined with other extraction techniques, enhancing the efficiency and reducing the processing time and costs [68,95] (Table 1).

The use of microwave energy (solvent-free microwave extraction (SFME) and microwave-assisted extraction (MAE)) shares similar advantages as the previous cases: a reduction of costs, extraction times, energy consumption and CO_2_ emissions [96]. A microwave reactor is the source of heat that promotes the bursting and release of accumulations of essential oils [97]. It represents a more efficient method for extraction of essential oils from the *Zingibereaceace* family, because it is able to reduce the extraction time from four h in hydrodistillation to one h, avoiding the formation of degradation products and obtaining the maximum yield [97]. Furthermore, the use of microwave extraction gives rise to other categories of techniques to improve its performance, such as hydrodistillation assisted by microwave (HDAM), steam distillation assisted by microwave (SDAM), vacuum microwave hydrodistillation (VMHD) or microwave by hydrodiffusion and gravity (MHG) [96,98] (Table 1).

Finally, solvent extraction was also used for the extraction of *C. longa* essential oil. It overcomes the problem of excessive heat reached with certain conventional techniques and, consequently, avoids the loss of the compounds and properties of the essential oil [99]. Ethanol, hexane or chloroform are some of the solvents used to extract turmeric essential oil, being the last one with which a higher yield of turmeric essential oil was obtained [100]. Recently, a group of researchers proposed freons as suitable and safe extractants of the essential oil from the roots of *C. longa* and its main components [101] (Table 1).

In general, the result is a yellow to orange-coloured liquid having a fresh, peppery and aromatic odour with sweet orange and ginger notes and a sharp and burning bitter taste [63,64]. These physical characteristics, as well as the chemical composition and related properties of the essential oil, may vary depending on the extraction technique. For this reason, the selection of both the most adequate method and operating conditions is key to obtain the maximum amount and quality of the *C. longa* essential oil [17]. Together with the extraction method, other factors such as drying and storage processes also influence the chemical composition of turmeric essential oil, being necessary the subsequent identification of the chemical composition to identify the variations and the quality control of turmeric essential oil.

## 3. Chemical Analysis of the Essential Oil Obtained from *C. longa* Rhizomes

The chemical composition of the essential oil obtained from *C. longa* rhizomes has been widely determined through gas chromatography-mass spectrometry (GC-MS) (Table 2), which is normally used for a sesquiterpenoid analysis [102] alone or combined with gas chromatography-flame ionisation detector (GC-FID) [103,104,105] to achieve a quantitative analysis. The determination of the chemical composition is key, because the components of the essential oil and their concentration can be considered a fingerprint conferring specific characteristics and properties [106].

**Figure 1:**
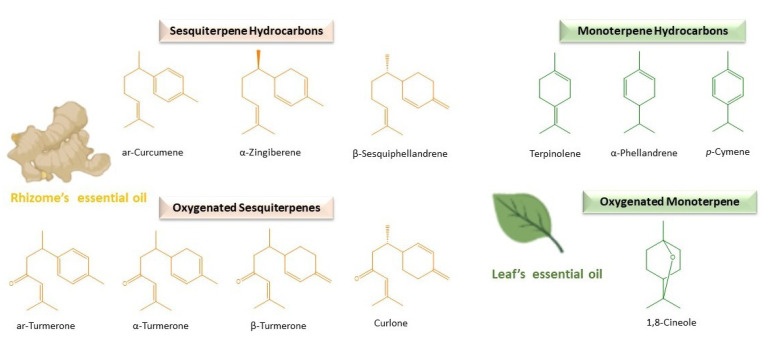
Main compounds found in the rhizomes and leaves of turmeric essential oils.

As a general rule, oxygenated sesquiterpenes have been identified as the predominant ones (Table 2) and the principal reason of the biological activity of turmeric essential oil [107]. Concretely, turmerones (α-, β- and ar-) represent the major and the most distinctive individual components [108,109] (Table 2 and Figure 1). They give interesting properties to *C. longa* essential oil, such as anticancer, anti-inflammatory, antioxidant and the prevention of dementia [72,110,111,112,113]. Even they enhance the bioavailability and activity of other important turmeric components like curcumin [114,115,116]. In particular, ar-turmerone (6S-2-methyl-6-(4-methylphenyl) hept-2-en-4-one) has been identified as the leading one, followed by α- and β-, in *C. longa* rhizome oil (Table 2). Many authors have reported about the therapeutic potential of ar-turmerone and its numerous benefits for human health [113]. Lee demonstrated its antibacterial activity against human pathogens like *Clostridium perfringens* and *Escherichia coli* [117]. In the same year, he also reported a higher inhibitory effect than aspirin in platelet aggregation induced by collagen and arachidonic acid [118]. Other researchers have proposed ar-turmerone as a natural anticancer and cancer-preventive agent, being considered the α,β-unsaturated ketone of the molecule, the principal pharmacophore, for this activity [51,119,120,121]. ar-Turmerone has also been observed as useful in the prevention and attenuation of inflammatory diseases like psoriasis and neuronal ones [122,123].

Oxygenated sesquiterpenes also constitute the predominant group in the essential oils obtained from the rhizome of other species included in the genus *Curcuma* [124]. For instance, curzerenone was the main compound in the rhizome oil of *C. angustifolia* and *C. zedoaria*; curdione was the major one in *C. nankunshanensis*, *C. wenyujin* and *C. kwangsiensis*; germacrone in *C. sichuanensis* and *C. leucorhiza*; β-elemenone in *C. nankunshanensis* var. *nanlingensis*; xanthorrhizol in *C. xanthorrhiza* and velleral in *C. attenuata* [124,125,126,127,128]. Turmerones are normally present, being considered the most representative components in general. Nevertheless, their amount may vary between species, probably due to the intrinsic differences between them [129]. The quantification of oxygenated sesquiterpenes, together with the identification of the secondary components, are key for the distinction and quality control of *Curcuma* spp. [17,130].

The sesquiterpenoids are generally followed by smaller quantities of sesquiterpene hydrocarbons in *C. longa* rhizome oil (Table 2 and Figure 1). This group is characterised by great structural diversity, providing a variety of fragrances and characteristic aromas to the essential oil [131]. Specifically, monocyclic bisabolane derivatives with a C_6_-ring formed in analogy to the menthane skeleton highlighted in turmeric essential oil obtained from rhizomes. Some examples are bisabolene isomers (β-bisabolene), α-zingiberene and ar-curcumene, characteristic of *Curcuma* spp. and ginger. β-caryophyllene is also common, widely spread in food plants and derived from α-humulene, with a C_9_-ring fused to a cyclobutane ring [132]. Sesquiterpene hydrocarbons predominate over oxygenated ones in the rhizome oil of other *Curcuma* spp., such as *C. aromatica* (Sesquiterpene Hydrocarbons (SH): 8.30% ± 1.90% and Oxygenated Sesquiterpenes (OS): 7.10% ± 2.14%) and *C. kwangsiensis* var *nanlingensis* (SH: 9.76% ± 1.89% and OS: 6.80% ± 1.27%) [124].

The amount of monoterpene hydrocarbons and oxygenated monoterpenes are usually lower in most samples of rhizome essential oil of *C. longa* (Table 2). Contrarily, they constitute the most abundant group in the rhizome oil of other different *Curcuma* spp., such as *C. amada* [133], as well as in the essential oils obtained from the aerial parts of *C. longa* [17,134,135,136,137]. Regarding this, the yield of *C. longa* essential oil varied between the leaves (23%), rhizomes (48%) and rhizoids (27%), and the chemical composition was different between the leaf petiole, lamina and rhizoid oils (myrcene, *p*-cymene, etc.) compared to the stem and rhizome ones in which turmerones predominated [138]. α-Phellandrene, terpinolene and 1,8-cineole (Figure 1) are usually the most abundant compounds detected in the essential oil extracted from the leaves of *C. longa* [36,39,43,44], whereas turmerones are found in minor concentrations (Table 2) [109], being also usually found in the essential oils of the aerial parts of *C. longa p*-cymene, α-terpinene, myrcene and pinenes (Table 2) [134,135,137,139,140]. However, in samples of *C. longa* grown in Nigeria, the leaf essential oil was dominated by turmerones, like in rhizomes (Table 2) [141,142]. In addition, important concentrations of C_8_-aldehyde (20.58%) were found in the essential oil of *C. longa* leaves in a high-altitude research station in Odisha, India [140]. The concentration of these compounds can be increased by enhancing the leaf biomass production [143].

The aerial parts of *C. longa* normally end as waste products. An interest approach is their recycling to obtain biologically active compounds. In this sense, *C. longa* leaf essential oil and its principal component α-phellandrene have demonstrated remarkable insecticidal activity against *Cochliomya macellaria*, causative agents of myasis in humans and animals, as well as against *Lucilia cuprina* [144,145], being also a *C. longa* leaf essential oil highlight because of its medicinal and food-preservation properties, with a significant inhibition of microbial growth and toxin production [146,147].

On the other hand, several studies corroborate that the qualitative and quantitative chemical compositions of turmeric rhizomes essential oil may fluctuate according to many factors [124,148,149]. Sometimes, different chemical compositions come from the intrinsic characteristics of each genotype. In fact, certain traits of a specific variety of *C. longa* can influence the content of rhizome oil, representing good criteria for the selection of high-yield ones. Regarding this, an interesting study observed a direct relationship between plant height and rhizome oil content, as well as a negative correlation between the amount of essential oil in the dry leaf with the one contained in the fresh rhizome [150]. A clear example of genotype influence is the dissimilar chemical composition between yellow *C. longa* rhizome oil rich in oxygenated sesquiterpenes (ar-turmerone, turmerone, curlone, etc.) and red one with oxygenated monoterpenes (carvacrol, citral, methyl eugenol, geraniol, etc.) as principal compounds more similar to *Origanum* or *Thymus* spp. [151]. Indeed, the rhizome colour is closely related to the beneficial properties of *C. longa* [152]. The influence of the genotype or cultivars have also been reported by other authors who observed significant variations in the yield and chemical composition of rhizome oils of *C. longa* under similar climatic conditions [153,154,155].

Together with the genetic and environmental factors, the geographic location contributes to the different yields and quality of *C. longa* rhizome oils, even developing different chemotypes [39,109]. In India, the region of production determines the type of turmeric [156]. Samples from Nepal included α- and β-turmerones (8.19% and 17.74%, respectively) between other compounds like *epi*-α-patshutene (7.19%), β-sesquiphellandrene (4.99%), 1,4-dimethyl-2-isobutylbenzene (4.4%), (±)-dihydro-ar-turmerone (4.27%) and zingiberene (4.03%) [33]. The main components of the essential oil from Nigeria were ar-turmerone, α-turmerone and β-turmerone [141,157], while turmerones (approximately 37%), together with terpinolene (15.8%), zingiberene (11.8%) and β-sesquiphellandrene (8.8%), predominated in the rhizome oil from Reunion Island [134]. Turmerones still are also the predominant compounds in samples from Faisalabad (Pakistan) and Turkey [104,158]. In the South American continent, the essential oil isolated from rhizomes grown in Ecuador was rich in ar-turmerone (45.5%) and α-turmerone (13.4%), similar to Colombian samples, while that from Brazil was dominated by zingiberene (11%), sesquiphellandrene (10%), β-turmerone (10%) and α-curcumene (5%) [105,107,159].

The analysis of each *C. longa* habitat’s conditions can help to predict the features of the resulting essential oil and enhance its yield and quality; what results especially important for its optimisation and commercialisation. Altitude, humidity, rainfall, temperature, soil pH, organic carbon, nitrogen, phosphorous and potassium are some of the factors that lead to wide variations in the yield and chemical composition of rhizome essential oil. From the development of predictive models and in vivo tests, the altitude, soil pH, nitrogen and organic carbon have been observed as enhancers of rhizome essential oil production. Amongst them, nitrogen and organic carbon raise the turmerone content concretely and phosphorous and potassium the oil yield [40,160,161,162]. Land configurations involving furrows and thatches surrounding *C. longa* reduce the loss of these soil nutrients, enhancing the rhizome yield [41].

The stage of maturity of *C. longa* rhizomes can also influence in the yield, chemical composition and properties of the essential oil. In relation to this, Garg et al. demonstrated that the percentage of the essential oil content widely varied between fresh and dried rhizomes of 27 accessions of *C. longa* in North India [163]. Similarly, Sharma et al. also observed certain variations in the qualitative and quantitative chemical compositions between the essential oils extracted from a mix of 5–10 month-old rhizomes and eight ones [139]. Furthermore, Singh et al. confirmed that fresh rhizome essential oil contained a major quantity of the active compound turmerone than dry ones, consequently having stronger activity [164]. A different trend was observed by Gounder et al., who reported the higher activity of cured (fresh rhizome boiled in water, dried in shade and polished) and dried rhizome oils over fresh ones [165], probably due to the lower percentage of ar-turmerone and β-turmerone. Anyway, the control of the drying conditions constitutes an important parameter in order to obtain the highest content of essential oil in the minimum time possible [166,167]. The sun and mechanical drying coexist as drying methods of *C. longa* rhizomes [156]. In particular, Monton et al. confirmed that one hour of microwave drying without conventional drying represented the optimum conditions to obtain the highest content of turmeric essential oil [167].

**Table 2:** Main components of *C. longa* essential oil according to the part of the plant used, origin, method of extraction and analysis. GC-MS: gas chromatography-mass spectrometry, CG-FID: flame ionisation detector, SFE: supercritical fluid extraction, SWE: supercritical water extraction and: CG-FTIR: gas chromatography-Fourier-transform infrared.

Part of Turmeric	Origin	Method of Extraction	Analysis	Yield	Main Components	Ref.
Powdered rhizomes	Nepal	Hydrodistillation Clevenger	GC-MS	3.0%	β–turmerone (17.74%), α-turmeron (8.19%), *epi*-α-patschutene (7.19%), β–sesquiphellandrene (4.99%), 1,4-dimethyl-2-isobutylbenzene (4.4%)	[33]
Pulverized rhizome	India	Steam distillation + vacuum distillation	GC-MS	1.6–46.6%	Turmerones, *l*-zingiberene, β–sesquiphellandrene, ar-curcumene	[72]
Rhizomes	Brazil	Hydrodistillation assisted by microwave (HDAM)	GC-MS	0.6%	ar-turmerone (50.37 ± 0.99%), β–turmerone (14.39 ± 0.33%), ar-curcumene (6.24 ± 0.21%)	[98]
Rhizomes	Brazil	HDAM + Cryogenic grinding (CG)	GC-MS	1.00%	ar-turmerone (47.97 ± 1.19%), β–turmerone (13.70 ± 0.55%), ar-curcumene (5.94 ± 0.27%)	[98]
Rhizomes	Brazil	Steam distillation assisted by microwave (SDAM)	GC-MS	0.9%	-	[98]
Rhizomes	Brazil	SDAM + CG	GC-MS	1.45%	-	[98]
Powdered dried rhizome	Serbia	Hydrodistillation Clevenger	GC-MS and GC-FID	0.3 cm^3^/100 g	ar-turmerone (22.7%), turmerone (26%) and curlone (16.8%)	[104]
Rhizomes	Pakistan	Hydrodistillation	GC-MS	0.673%	ar-turmerone (25.3%), α-turmerone (18.3%) and curlone (12.5%)	[158]
Powdered rhizomes	Thailand	Hydrodistillation Clevenger	GC-MS	-	ar-turmerone (43–49%), turmerone (13–16%) and curlone (17–18%)	[166,167]
Dried rhizomes	Brazil	SFE	GC-MS	0.5–6.5 g/100 g	ar-turmerone (20%) and ar-, α- and β–turmerones (~75%)	[91]
Dried rhizomes	Brazil	Extraction with volatile solvents	GC-MS and CG-FID	5.49%	α-turmerone and β –turmerone (~8.7%), ar-turmerone (~3.6%)	[104]
Dried rhizomes	Brazil	Steam distillation	GC-MS and CG-FID	0.46%	ar-turmerone (~12.8%), α-turmerone and β –turmerone (~4.1%)	[104]
Dried rhizomes	China	Steam distillation	GC-MS	4.50% *w/w*	ar-turmerone (11.81%)	[124]
Dried rhizomes	Nigeria	Hydrodistillation Clevenger	GC-MS	1.33% *w/w*	ar-turmerone (44.4%), α-turmerone (20.8%), β–turmerone (26.5%)	[141]
Dry rhizomes	India	Hydrodistillation Clevenger	GC-MS	2.9%	ar-turmerone (21.4%), α-santalene (7.2%) and ar-curcumene (6.6%)	[164]
Dried rhizomes	India	Hydrodistillation Clevenger	GC-MS	3.05 ± 0.15%	ar-turmerone (30.3%), α-turmerone (26.5%), β–turmerone (19.1%)	[167]
Cured rhizomes	India	Hydrodistillation Clevenger	GC-MS	4.45 ± 0.37%	ar-turmerone (28.3%), α-turmerone (24.8%), β–turmerone (21.1%)	[167]
Dried root	-	SFE	GC-MS	2–5.3 wt%	ar-turmerone (31–67.1%), β–turmerone (2–37.9%), α-turmerone (0–21.3%)	[87]
Fresh rhizomes	Brazil	Hydrodistillation Clevenger	GC-MS	1000 µL	α-turmerone (42.6%), β –turmerone (16.0%) and ar-turmerone (12.9%)	[34]
Fresh rhizomes	India	Hydrodistillation Clevenger	GC-MS	0.6–2.1%	Turmerone (35.24–44.22%)	[39]
Fresh rhizomes	India	Hydrodistillation Clevenger	GC-MS	0.8%	α-turmerone (44.1%), β–turmerone (18.5%) and ar-turmerone (5.4%)	[43]
Fresh rhizomes	India	Hydrodistillation Clevenger	GC-MS	0.36%	ar-turmerone (31.7%), α-turmerone (12.9%), β–turmerone (12.0%) and (Z)- β–ocimene (5.5%)	[44]
Fresh rhizomes	India	Modified distillation process	GC-MS	2.09–2.50%	ar-turmerone (45.27%), curlone (5.6%), turmerone (4.4%), zingiberene (4.01%), ar-curcumene (4.01%), dehydrocurcumene (2.0%)	[73]
Fresh rhizome	Malaysia	SFE	GC-MS	-	ar-turmerone (10.84–21.50%), turmerone (36.14–45.68%) and curlone (21.27–22.30%)	[79]
Fresh rhizomes	Iran	SWE	GC-MS	0.98%	ar-turmerone (62.88%), curcumin (10.49%), β–sesquiphellandrene (9.62%), α-phellandrene (6.50%)	[86]
Fresh rhizomes	Ecuador	Steam distillation	GC-FID and GC-MS	0.8% *v/w*	ar-turmerone (45.5%) and α-turmerone (13.4%)	[105]
Fresh rhizomes	France	Steam distillation	GC-MS and GC-FTIR	1.1%	α-turmerone (21.4%), zingiberene (11.8%), terpinolene (15.8%), β–sesquiphellandrene (8.8%), ar-turmerone (7.7%) and β–turmerone (7.1%)	[134]
Fresh mature rhizomes	Bhutan	Hydrodistillation Clevenger	GC-MS	2–5.5%	α-turmerone (30–32%), ar-turmerone (17–26%) and β–turmerone (15–18%)	[139]
Fresh rhizome	India	Steam distillation	-	2.03–6.50%	-	[156]
Fresh rhizomes	India	Hydrodistillation Clevenger	GC-MS	1.8–3.73 mL/plant	ar-turmerone (39.5–45.5%), curlone (9.8–11.7%), α-phellandrene (5.5–7.7%), eucalyptol (3.2–5.5%), β–himachalene (1.6–5.5%) and copen-11-ol (2.3–5.4%)	[157]
Fresh rhizomes	Nigeria	Hydrodistillation Clevenger	GC-MS	10.5 g (0.7% *w/w*)	Turmerone (35.9%), α-phellandrene (15.5%), curlone (12.9%), 1,8-cineole (10.3%) and ar-turmerone (10.0%)	[157]
Fresh rhizomes	Brazil	Hydrodistillation Clevenger	GC-MS	0.70%	Zingiberene (11%), sesquiphellandrene (10%), β–turmerone (10%) and α-curcumene (5%)	[159]
Mature fresh rhizomes	India	Hydrodistillation Clevenger	GC-MS	1.4%	ar-turmerone (24.4%), α-turmerone (20.5%) and β–turmerone (11.1%)	[166]
Fresh rhizomes	India	Hydrodistillation Clevenger	GC-MS	3.52 ± 0.23%	α-turmerone (33.5%), ar-turmerone (21.0%), β–turmerone (18.9%)	[167]
Semi dried leaves and fresh rhizomes	India	Continuous water circulation with steam distillation	GC-MS	Leaves: 2.75–2.83% Rhizomes: 2.38–2.48%	Rhizome: Bisabolene (0.4%), ar-curcumene (2.3%), zingiberene (4.01%), dehydrocurcumene (2.0%), ar-turmerone (15.8%), turmerone (4.4%) and curlone (5.6%)	[71]
Semi-ripened and dried leaves	India	Water distillation techniques	GC-MS	0.25–0.28% *v/w*	Terpinolene (33.0–57.6%), 1,8-cineole (1.9–7.9%), α-terpinene (1.7–3.9%), α-phellandrene (1.4–3.1%)	[36]
Partially senescenced leaves	India	Hydrodistillation Clevenger	GC-MS	-	α-phellandrene, *p*-cymene, α-terpinolene, 1,8-cineole, *p*-cymen-8-ol	[137]
Dried leaves	Nigeria	Hydrodistillation Clevenger	GC-MS	0.67% *w/w*	ar-turmerone (63.4%), α-turmerone (13.7%), β–turmerone (12.6%)	[141]
Dried leaves	Nigeria	Hydrodistillation Clevenger	GC-MS	0.67% *w/w*	ar-turmerone (63.4%), α-turmerone (13.7%), β–turmerone (12.6%)	[142]
Leaves	Bhutan	Hydrodistillation Clevenger	GC-MS	0.3–0.42%	α-phellandrene (18.2%), 1,8-cineole (14.6%) and *p*-cymene (13.3%)	[139]
Leaves	India	Hydrodistillation Clevenger	GLC	1.32%	α-phellandrene (38.24%), C8-aldehyde (20.58%), 1,8-cineole (8.64%), α-pinene (2.88%) and β–pinene (2.36%)	[140]
Leaves	Pakistan	Hydrodistillation Reverse dean-stark method	GC-MS	145%	Eucalyptol (10.27%), β–pinene (3.57%), 2-methylisoborneol (2.91%), limonene (2.73%), β–phellandrene (2.49%)	[143]
Fresh leaves	India	Hydrodistillation Clevenger	GC-MS	0.2–1.9%	α-Phellandrene (30.82–39.85%), terpinolene (25.74–26.59%) and eucalyptol (7.52–7.66%)	[39]
Fresh leaves	India	Hydrodistillation Clevenger	GC-MS	0.65%	α-phellandrene (53.4%), terpinolene (11.5%) and 1,8-cineole (10.5%)	[43]
Fresh leaves	India	Hydrodistillation Clevenger	GC-MS	0.53%	α-phellandrene (9.1%), terpinolene (8.8%), 1,8-cineole (7.3%) and undecanol (7.1%)	[44]
Roughly crushed fresh leaves	France	Steam distillation	GC-MS and GC-FTIR	0.5%	Terpinolene (77%), 1,8-cineole (4.6%), α-terpinene (3.7%), α-phellandrene (2.8%), myrcene (1.4%) and δ-3-carene (1.1%)	[134]
Fresh leaves	India	Steam distillation	GC-MS	0.15%	Terpinolene (71.2%), 1,8-cineole (6.2%), *p*-cymen-9-ol (4.2%)	[136]
Fresh leaves and stems	Colombia	Steam distillation	GC-MS	-	Turmerone (36.9%), α-turmerone (18.9%) and β–turmerone (13.6%)	[107]
Fresh aerial parts	India	SFE	GC-MS	2.8%	*p*-cymene (25.4%), 1,8-cineole (18%), *cis*-sabinol (7.4%), β–pinene (6.3%)	[135]
Roughly crushed fresh flowers	France	Steam distillation	GC-MS and GC-FTIR	0.15%	Terpinolene (67.4%), 1,8-cineole (4.6%), α-terpinene (4.4%), α-phellandrene (3.6%), myrcene (2%) and zingiberene (1.3%)	[134]

*C. longa* nutrition also has a significant impact in the yield and composition of rhizome oil. Especially, fertilizer use can enhance the productivity of volatile oil of *C. longa* rhizomes 6% [148]. Furthermore, a prior treatment with minerals during in vitro rhizome development followed by a fertilizer treatment in a greenhouse increases the percentage of volatiles in *C. longa* rhizomes. Particularly remarkable is the interaction of KNO_3_ and Ca^2+^, which favours the accumulation of sesquiterpenes in turmeric rhizome [168]. An interesting research proposed the use of arbuscular mycorrhizal fungi instead of chemical fertilizers in the cultivation of *C. longa* rhizomes. These optimise the absorption of nutrients and water, augment the metabolic activity of the plant, etc. In consequence, the root system becomes more robust, and the chemical composition of the essential oil is improved, increasing the production of certain compounds, including caryophyllene, α-curcumene, β-bisabolene and β-curcumene, using sustainable technologies [169,170]. Finally, the postharvest management of turmeric rhizomes also has a noteworthy influence on the quality of the derived products. Concretely, the boiling conditions, way of slicing, type of mill and speed of crushing and presence of heat and oxygen need to be controlled and standardised to obtain essential oils with certain characteristics [156].

In conclusion, the study of the chemical composition of the essential oil from the rhizome of *C. longa* gives us an idea of the characteristics and possible properties that it possesses. Sesquiterpenes are usually the main compounds in *C. longa* rhizome essential oil, highlighting the oxygenated turmerones followed by sesquiterpene hydrocarbons (Figure 1). However, the qualitative and quantitative chemical compositions of the essential oil can vary depending on the genetic and commented on factors. The knowledge of these can help to achieve a high-yield product with useful composition and properties for the agri-food industry.

## 4. Potential Applications of *C. longa* Essential Oil Obtained from Rhizomes in the Agri-Food Industry

**Figure 2:**
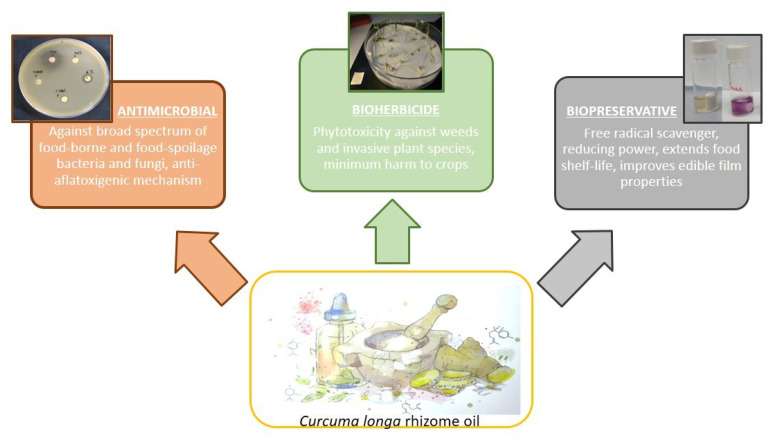
Representation of the roles that *Curcuma longa* rhizome oil can play in the safer and more sustainable emerging agri-food industry: antimicrobial, herbicidal and antioxidant activities.

Foodborne diseases, spoilage, insect and weed infestation are some common problems that cause significant economic losses to the agri-food industry. Chemical preservatives and pesticides have been widely exploited to maintain and enhance yields and productivity. However, the numerous handicaps derived from their overuse have been extensively described. As a result, sustainability has become an increasingly important subject in the agri-food industry. The characteristics of certain natural products, especially essential oils (zero waste), have become a matter of study as sustainable alternatives [171,172,173,174,175,176]. Amongst them, *C. longa* rhizome oil can take part in the safer and eco-friendly emergent agri-food industry due to its promising antimicrobial, herbicidal and antioxidant activities (Figure 2).

### 4.1. Prevention and Inhibition of Microbial Attack in Crops and Food-Spoilage Microorganisms

Microbial contamination can affect any step of the food chain, from seed germination to food processing and storage [177,178,179,180]. Initially, the seed-borne pathogens endanger the correct development of grains, affecting both yield and quality [179]. Besides, bacteria and fungi are the principal causative agents of many postharvest diseases. *Erwinia*, *Pseudomonas*, *Corynebacterium*, *Aspergillus* and *Fusarium* are some of the most common food spoiler species, seriously compromising the quality of food products. Moreover, human health may be disturbed by the contact with these contaminated products [181,182], along with a deleterious impact in the reliability and economics of the agri-food industry [177,183,184]. Synthetic pesticides and preservatives, some of them with detrimental effects, have been the most commonly used formulations to prevent and stop the growth of these microorganisms. Therefore, natural, safer and eco-friendly antimicrobials are demanded by the consumers [178,180,185,186]. Essential oils constitute a potential alternative, because they possess antimicrobial activity, individually or in combinations between them and with antibiotics. They prevent food deterioration, maintain their appearance and quality and are able to be used in biopreservation and biocontrol in the agri-food industry [187,188,189,190,191].

In general, the essential oils proceeding from the rhizomes of the genus *Curcuma* have demonstrated noteworthy antimicrobial activity [192,193,194]. Amongst them, the essential oil of *C. longa* rhizome with 58% of ar-turmerone, together with limonene and borneol as the principal compounds, has presented a dose-dependent antimicrobial activity against a broad spectrum of food-borne and food-spoilage bacteria and fungi, including *Bacillus subtilis*, *Salmonella choleraesuis*, *Escherichia coli*, *A. niger* and *Saccharomyces cerevisiae* but at higher doses than the traditional chloramphenicol and amphotericin antibiotics [195] (Table 3). The addition of *C. longa* essential oil (33.42% ar-turmerone, 22.35% α-turmerone and 20.14% β-turmerone) to an edible film with sorbitol and egg white protein power improved both the properties of the film (thickness and lipophilicity) and its antibacterial activity against *E. coli* and *Staphylococcus aureus* [196] (Table 3).

Usually, bacterial contamination is more difficult to detect, because food generally appears normal until advanced infection. In contrast, fungal contamination can be easily perceived, as it normally alters the odour, appearance and texture of food [177,178,197]. *C. longa* essential oil has already demonstrated its strong fungicidal effect against the causal agents of important diseases in crops [192,198]. In particular, the radial growth of *Colletotrichum gloeosporioides*, *Sphaceloma cardamomi* and *Pestalotia palmarum* were completely inhibited after the treatment with essential oil from *C. longa* rhizomes at 1–5%. Other phytopathogenic fungi, such as *Rhizoctonia solani*, *Aspergillus* sp. and *Fusarium* sp., were also notably affected, especially at the highest concentration (5%) assayed [199] (Table 3).

It is interesting to note that essential oils represent a natural alternative to the usually employed weak-acid preservatives in the prevention of *A. niger,* a common contaminant of yogurt, ready-to-drink beverages and, especially, bakery products [200,201]. Particularly, packaging with a biopolymer film containing turmeric essential oil (35.46% turmerone, 20.61% cumene and 13.82% ar-turmerone) constitutes a sustainable and efficient technology to protect these food products against attacks of the filamentous fungus. The biopolymer film acts as a carrier, releasing in a sustained way the antimicrobial agent turmeric essential oil [202] (Table 3). In fact, the addition of turmeric essential oil in edible coating films could enhance food protection from microbial contamination in general. In relation to this, the fungal growth of common spoilers of pumpkin *Penicillium* and *Cladosporium* spp. were reduced 60.3% and 41.6%, respectively, for 15 days with an edible coating based on achira starch (*Canna indica* L.) containing 0.5% *w/w C. longa* rhizome oil [203] (Table 3).

The antifungal effect of *C. longa* essential oil has been tested in other *Aspergillus* spp., such as *A. flavus*, a common contaminant of cereals, legumes, juices, and fresh and dried fruits [182,204,205,206,207,208], as well as one of the major source of aflatoxins in agricultural crops, considered the most problematic mycotoxins worldwide [181,209,210]. The growth rate of *A. flavus* was significantly reduced with only 0.10% *v/v* of *C. longa* rhizome oil (33.2% ar-turmerone, 23.5% α-turmerone and 22.7% β-turmerone). Furthermore, the germination and sporulation were completely inhibited at 0.5% *v/v* [211] (Table 3).

Regarding *Fusarium* spp., versatile spoilers of fruit, vegetables, cereals, etc. [212,213] generating important economic losses in the agri-food industry, *C. longa* rhizome essential oil has exhibited also promising results. The mycotoxin production, particularly of thrichothecenes and fumonisins, with serious health impacts in humans and livestock by their potentially carcinogenic and inhibition of the protein synthesis, respectively [214], is another problem to solve. The essential oil obtained from the fresh rhizomes of *C. longa* (42.6% α-turmerone, 16.0% β-turmerone and 12.9% ar-turmerone) significantly affected the development of *F. vericillioides* by decreasing the thickness and length of the microconidia, as well as the fungal biomass. The fumonisin production was also significantly inhibited [34] (Table 3). Likewise, *C. longa* rhizome oil (53.10% ar-turmerone) had a considerable effect in the morphology of the mycelia and spores, as well as in the zearalenone production of *F. graminearum* [215], being the mycelial growth of *F. moniliforme* and *F. oxysporum* inhibited at 1000 and 2000 ppm, respectively [192] (Table 3).

On the other hand, the essential oil obtained from other parts of *C. longa* with different chemical compositions has also shown antimicrobial activity. In this sense, *C. longa* essential oil dominated by oxygenated monoterpenes (82.0%) displayed promising in vivo antifungal activity against *P. expansum* and *Rhizopus stolonifer* when combined with *A. sativum* essential oil, representing a natural alternative to chemical fungicides in tomato protection [216]. Similarly, *C. longa* essential oil rich in monoterpenes (20.4% α-phellandrene, 10.3% 1,8-cineole and 6.19% terpinolene) and with considerable quantities of α- and β-turmerone (19.8% and 7.35%) presented one of the highest MICs (0.06–0.36 mg/mL) with respect to 11 different essential oils against five food-spoilage yeasts [217] (Table 3).

Therefore, the high antimicrobial activity of *C. longa* essential oil may be due to a synergism between the usual main compounds ar-turmerone, turmerone and curlone and the other phenolic group [218].

Regarding these data, the essential oil from the rhizome of *C. longa* can be considered a green alternative for biopreservation in the agri-food industry. It has demonstrated promising dose-dependent antimicrobial activity against a wide range of microorganisms. This efficacy is not always shared with the essential oils extracted from other parts of *C. longa*. Therefore, its efficacy may be due to its particular chemical composition, especially to the predominance of turmerones and combinations with other oxygenated compounds. This makes *C. longa* rhizome oil the subject of incorporation in edible coating films and other encapsulating technologies for future applications.

### 4.2. Herbicidal Activity

The resistance and tolerance development of weeds, crop damage or environmental pollution are the main problems due to the continuous use of synthetic herbicides in global agriculture [219,220,221]. Alternatives to synthetic herbicides for weed management and food security require the research of natural sources such as essential oils to develop safer and more sustainable herbicides without significantly affecting crops yields. Several essential oils have demonstrated promising herbicidal properties, inhibiting seed germination and/or seedling growth of a broad number of weeds [175,222,223,224,225]. In fact, some of them are already the main components of several commercial herbicidal compositions, taking part in the construction of a harmless and eco-friendlier emergent agri-food industry [226,227].

Regarding turmeric, the rhizome essential oil (38.7% ar-turmerone, 18.6% β-turmerone and 14.2% α-turmerone) has proven to be a potential post-emergent treatment in the control of weeds such as common purslane (*Portulaca oleracea* L.), especially aggressive in agriculture because of its versatility in affecting a wide variety of scenarios due to its tolerance to changes and rapid growing [228,229], Italian ryegrass (*Lolium multiflorum* Lam.), rapidly growing weed with the capacity of producing large quantities of seeds, being particularly competitive in small grain and vegetable harvests, where it represents a great problem due to the development of herbicide resistance [230,231] and barnyard grass (*Echinochloa crus-galli* (L.) Beauv.), considered one of the world’s worst weeds infesting cropping systems [232], especially detrimental in rice paddies, where it interferes with canopy light transmission, triggering a series of metabolic alterations in rice that can lead to severe losses of even 55.2% [233]. Concretely, it reduced the hypocotyl development of the three weeds 56.55%, 40.45% and 39.33%, respectively, from 0.125 to 1 µL/mL, without affecting either the seed germination or the hypocotyl growth of the tomato, cucumber and rice crops [234] (Table 3).

The harmlessness of *C. longa* rhizome essential oil for food crops, a great challenger in the search for natural herbicides, has been corroborated by other authors. For instance, Prakash et al. confirmed that it did not affect the germination of chickpea seeds. The mean length of both hypocotyl and radicle were not significantly reduced after three days of exposure to the essential oil regarding control (3.65 and 0.82 cm *vs.* 3.75 and 0.93 cm, respectively). Only its combination with *Z. officinale* essential oils showed certain phytotoxicity against the seeds, probably due to the activity of *Z. officinale* [235]. However, the essential oil proceeding from other species included in the genus *Curcuma* have shown phytotoxic actions against food crops. For instance, *C. zedoaria* essential oil with a predominance of oxygenated compounds (18.20% *epi*-curzerenone and 15.75% 1,8-cineole) severely depressed the germination, germination rate and seedling development of lettuce and tomatoes. Particularly, the seed germination of both crops decreased from 80% to 0% and from 100% to 40%, correspondingly, at the highest dose of *C. zedoaria* essential oil (1.00%) assayed, and the hypocotyl and radicle growths were significantly reduced, with the essential oil at only 0.73–0.86% [236].

Furthermore, *C. longa* rhizome oil constitutes a potential candidate for biological control of the emerging invasive alien plant species. Specifically, it is outstanding in the inhibitory effect in the development of pampas grass (*Cortaderia selloana* (Schult. & Schult. f.) Asch. & Graebn.) and tree tobacco (*Nicotiana glauca* Graham.) from the lowest dose (0.125 µL/mL) assayed. Among them, *C. selloana* exhibited a special sensitivity to *C. longa* essential oil. The seed germination was drastically inhibited in a dose-dependent manner, achieving 81.71% of reduction at the highest dose (1 µL/mL) applied [234] (Table 3). It is interesting to note that the management of invasive species with sustainable alternatives is another important step in global agriculture, because these species are becoming naturalized in a wide number of areas with serious consequences: they influence the environment, change soil properties, affect diversity, etc. and, finally, are reverberating in socioeconomic factors, as well as human health [237,238,239].

On the other hand, other products derived from *C. longa* have demonstrated phytotoxic activity. In this way, the ethanolic extract completely inhibited the growth of the floating weed common duckweed (*Lemna minor* (L.) Griff.) at 100 and 1000 µg/mL [240], whereas the ethyl acetate extract (1000–10,000 ppm) showed the highest inhibitory effect vs. the seed germination and seedling growth of radishes in comparison to cyclohexane and *n*-hexane, which stimulated germination and elongation at 10,000 and 7500 ppm, respectively [241]; more recently, Akter et al. remarked on the potent inhibitory effect of the methanolic extract against the seed germination and seedling growth of both weed beggarticks (*Bidens pilosa* L.) and crops cress, radishes and lettuce. Especially, the major curcuminoids present in *C. longa*’s Ryudai gold variety strongly reduced the seed germination, as well as root and shoot growth of the weed (IC_50_ 8.7–12.9 and 15.5–38.9 µmol/L, respectively) [242].

Therefore, *C. longa* can be considered an important source of bioproducts with interesting phytotoxic properties. Especially, the rhizome essential oil has demonstrated apt herbicidal activity against specific weed and invasive plant species, without significantly harming food crops. These observations make the essential oil of *C. longa* rhizome a reference of investigation for new weedicide compounds. Further research involving more weed and crop species, as well as different conditions, is needed to keep demonstrating its potential as a bioherbicide.

### 4.3. Food Decay Prevention: Antioxidant Activity

Stored food products are subject to oxidation, involving a loss of quality, alteration of the organoleptic properties and nutritional value, as well as of food safety problems. Synthetic antioxidant additives commonly used to avoid this process are under controversy currently, which has led to an increased interest in the agri-food industry to use the preservative properties of plant products.

Several essential oils and their components have already demonstrated their potential role in overcoming storage losses and enhancing food shelf-lives in the near future [243,244,245]. Some have even been approved as flavour or food additives, and others are under validation. Nowadays, the encapsulation of essential oils is also being studied to try to stabilise their antioxidant activity and even enhance it [246].

In general, *C. longa* and its products have shown their antioxidant potential as biopreservatives of physicochemical and organoleptic properties of food items, such as paneer, white hard clams, rainbow trout, cuttlefish and mashed potatoes, either alone or in combination with other plant products. This property can be improved even more with the help of nanotechnology that may control the aqueous solubility and stability of turmeric derivatives [245,247,248,249,250,251,252,253,254,255].

The antioxidant properties of the turmeric essential oils have been widely studied. The leaf essential oil with 22.8% β-sesquiphellandrene and 9.5% terpinolene as the main compounds has been proposed as a potential option to prevent the oxidative deterioration of fat-containing food products because of its hydrogen-donating properties and reducing power [256] (Table 3). Likewise, *C. longa* rhizome oil is able to decrease lipid peroxidation and other processes related to free-radical formation, achieving the extending shelf-lives of food products. In fact, it has exhibited the lowest peroxide value with respect to oleoresins and synthetic antioxidants, meaning a more efficient inhibitory effect of the formation of the secondary oxidation product malondialdehyde [164] (Table 3). This effect has been corroborated by means of diverse methods that evaluate both the scavenging capacity for different free radicals and the metal ion-chelating ability of the essential oil. Particularly, the essential oil obtained from the fresh rhizomes of *C. longa* (α-turmerone (42.6%), β-turmerone (16.0%) and ar-turmerone (12.9%)) has exhibited satisfactory dose-dependent DPPH (2,2-diphenyl-1-picrylhydrazyl) and ABTS (2,2′-azino-bis-3-ethylbenzothiazoline-6-sulphonic acid) radical-scavenging activities (IC_50_ 10.03 and 0.54 mg/mL, respectively), as well as reducing power [34] (Table 3). Both the DPPH and ABTS methods are between the most carried out antioxidant capacity assays [257]. These are good estimators of the antioxidant activity of any extract in general, using a simple redox reaction between the antioxidant and reactive oxygen species (ROS), being considered the DPPH method as the first line for evaluating the ability of a compound and extract or other biological source to act as a free-radical scavenger or hydrogen donor because of its accuracy, simplicity and low cost [258]. On the other hand, ABTS has been observed as especially useful to track changes in the antioxidant system itself during the storage and processing steps [257]. Reducing power is usually a complementary test to the previous ones to further evaluate the antioxidant activity [259].

In this way, the antioxidant activity of *C. longa* rhizome essential oil stood out over 10 other different essential oils. Its free radical-scavenging potential was twice higher than that of Trolox (~60% vs. 28.2%, respectively), and the antioxidant activity (72.4%) was near the values of the reference essential oil *Thymus vulgaris* (90.9%) and butylated hydroxyanisole (BHA) (86.74%) [217] (Table 3). Similarly, the reducing potential of *C. longa* rhizome oil was also highlighted over *Eucalyptus* spp., such as *E. sideroxylon*, *E. tereticornis* and *E. citriodora* [130.5 ± 1.2, 122.1 ± 1.4 and 95.8 ± 1.0 µM ferric reducing antioxidant power (FRAP) equivalents, respectively], with 138.4 ± 1.1 µM FRAP equivalents. This value was even higher than the one of other *Curcuma* spp.—for instance, *C. aromatica* (130.6 ± 1.5 µM FRAP equivalents) [260]. This antioxidant potential has also been demonstrated in vivo. A starch/carboxymethyl cellulose (CMC) edible coating including *C. longa* oil suppressed the oxidase enzyme activity of fresh-cut “Fuji” apples by 9% [261] (Table 3).

The luminol-photochemiluminiscence (PLC) assay corroborated afterwards the high antioxidant activity of *C. longa* essential oil [217]. It results in an easy, fast and sensitive method to know the scavenging activity of antioxidants against the radical anion superoxide, especially for hydrophobic-like essential oils [217]. This property may be due to the total phenolic content of *C. longa* essential oil that also highlights over more than 15 essential oils from different plant species [235]. However, the phenolic compounds of *C. longa* essential oil and, consequently, the antioxidant activity can vary depending on the cultivation conditions. Especially, the substrate type, together with the presence of fungi, have significantly influenced the composition and activity of *C. longa* leaf essential oil [169,170]. The antioxidant activity can also change according to many other factors, such as the degree of dryness of *C. longa* rhizome. Specifically, the essential oil from the fresh rhizomes (24.4% ar-turmerone, 20.5% α-turmerone and 11.1% β-turmerone) exhibited higher DPPH radical scavenging, as well as Fe^2+^-chelating abilities, than the dry ones (21.4% ar-turmerone, 7.2% α-santalene and 6.6% ar-curcumene). The antioxidant activity of both essential oils was significantly higher than the commercial antioxidants BHA and butylated hydroxytoluene (BHT) [164] (Table 3). Nevertheless, other authors reported a different trend. Gounder et al. demonstrated throughout several tests that dried and cured rhizomes had higher antioxidant activity than the fresh ones (ar-turmerone (21.0–30.3%), α-turmerone (26.5–33.5%) and β-turmerone (18.9–21.1%)). Specifically, ABTS radical cation scavenging [Trolox equivalent antioxidant capacity (TEAC) 68.0, 66.9 and 38.9 µM at 1 mg/mL]; ferric-reducing antioxidant potential (TEAC 276.8, 264.1 and 178.4 µM at 1 mg/mL); total antioxidant capacity by phosphomolybdenum assay (686, 638 and 358 ascorbic acid equivalents per 1 mg of oil) and reducing power were stronger in dried and cured rhizome than in fresh ones, respectively [165]. These differences are mainly due to the different compositions reported by the authors [164,165] in the fresh and dry rhizome essential oils used in the test.

Several research carried out with turmeric rhizome essential oil without β-turmerone among the main compounds [105,262] reported lower DPPH bleaching potential and ferric-reducing antioxidant power of *C. longa* rhizome oil (45.5% ar-turmerone and 13.4% α-turmerone) principally, in comparison to those of Trolox (IC_50_ 14.5 ± 2.9 mg/mL *vs.* 0.012 ± 0.004 mg/mL and 389.0 ± 112.0 *vs.* 402.3 ± 20.1 µM ascorbic acid equivalents, respectively) [105], as well as negligible DPPH radical scavenging activity (38.7% ar-turmerone and 14.2% α-turmerone) with respect to other, different essential oils, among which were cinnamon, clove, green tea, lemon eucalyptus, rosemary, oregano and its main compound carvacrol [262] (Table 3).

**Table 3:** Antimicrobial, herbicidal and antioxidant activities of *C. longa* essential oil in the agri-food industry. DPPH: 2,2-diphenyl-1-picrylhydrazyl, ABTS: 2,2′-azino-bis-3-ethylbenzothiazoline-6-sulphonic acid, BHA: butylated hydroxyanisole and BHT: butylated hydroxytoluene.

**Antimicrobial Activity**			
**Chemical Composition**	**Concentration**	**Effect**	**Ref.**
42.6% α-Turmerone 16.0% β-Turmerone 12.9% ar-Turmerone	17.9 and 294.9 µg/mL	Decrease the development of *Fusarium verticillioides* by 56.0 and 79.3%, respectively, as well as the thickness and length of microconidia, fungal biomass and fumonisin production	[34]
51.8% ar-Turmerone 11.9% ar-Turmerol	1000 ppm	Complete mycelial growth inhibition of *Colletotrichum falcatum* and *F. moniliforme*	[192]
51.8% ar-Turmerone 11.9% ar-Turmerol	2000 ppm	Complete mycelial growth inhibition of *Curvularia pallescens*, *Aspergillus niger* and *F. oxysporium*	[192]
58% ar-Turmerone Limonene Borneol	>45–90 µg/disc	Significant inhibition of *Bacillus subtilis*, *Salmonella choleraesuis*, *Escherichia coli*, *A. niger* and *Saccharomyces cerivisiae* at higher doses than chloramphenicol and amphotericin	[195]
33.42% ar-Turmerone 22.35% α-Turmerone 20.14% β-Turmerone	1–2% (*v/v*)	Antibacterial activity against *E. coli* and *Staphylococcus aureus* when incorporated to an edible film with sorbitol and egg white protein	[196]
-	1–5%	Complete radial growth inhibition of *C. gloeosporoides*, *Sphaceloma cardamomi*, *Pestalotia palmarum, Rhizoctonia solani, Aspergillus* sp. and *Fusarium* sp.	[199]
35.46% Turmerone 20.61% Cumene 13.82% ar-Turmerone	>0.5 µL	Antifungal effect against *A. niger* when incorporated to a biopolymer film	[202]
-	0.5% *w/w*	Reduction of the growth of *Penicillium* and *Cladosporium* spp. in 60.3 and 41.6%, respectively, for 15 days when incorporated to an edible coating based on achira starch (*Canna indica* L.)	[203]
33.2% ar-Turmerone 23.5% α-Turmerone 22.7% β-Turmerone	0.10–0.5% *v/v*	Significant reduction of the growth rate of *A. flavus,* as well as complete inhibition of germination and sportulation	[211]
53.10% ar-Turmerone	2450 and 3300 µg/mL	Minimum inhibitory and minimum fungicidal concentration against *F. graminearum*	[215]
53.10% ar-Turmerone	3500 and 3000 µg/mL	Complete inhibition of fungal biomass and zearalenone production in *F. graminearum,* respectively	[215]
20.4% α-Phellandrene 19.8% α-Turmerone 10.3% 1.8-Cineole 7.35% β-Turmerone	0.06–0.36 µg/mL	One of the highest minimum inhibitory concentrations with respect to 11 different essential oils against five-food spoilage yeasts	[217]
**Herbicidal Activity**			
38.7% ar-Turmerone 18.6% β-Turmerone 14.2% α-Turmerone	0.125–1 µL/mL	Reduction of the hypocotyl growth of *Portulaca oleracea*, *Lolium multiflorum* and *Echinochloa crus-galli* in 56.55, 40.45 and 39.33%, respectively, without affecting neither seed germination nor hycopotyl growth of tomato, cucumber and rice crops	[234]
38.7% ar-Turmerone 18.6% β-Turmerone 14.2% α-Turmerone	1 µL/mL	Significant inhibition of *Cortaderia selloana* seed germination (81.71%)	[234]
38.7% ar-Turmerone 18.6% β-Turmerone 14.2% α-Turmerone	>0.125 µL/mL	Outstanding inhibitory effect in the development of *C. selloana* and *Nicotiana glauca*	[234]
**Antioxidant Activity**			
42.6% α-Turmerone 16.0% β-Turmerone 12.9% ar-Turmerone	IC_50_ 10.03 mg/mL (DPPH) IC_50_ 0.54 mg/mL (ABTS)	Dose-dependent DPPH and ABTS radical scavenging activities, as well as reducing power	[34]
45.5% ar-Turmerone 13.4% α-Turmerone	IC_50_ 14.5 ± 2.9 mg/mL (DPPH) 389.0 ± 12.0 µM Ascorbic Acid (AA) eq.	Low DPPH bleaching potential and ferric-reducing antioxidant power in comparison to Trolox	[105]
24.4% ar-Turmerone 20.5% α-Turmerone 11.1% β-Turmerone	<100 Meq/kg (peroxide value) 0.04–0.08 TBA value 5–20 µL (DPPH) 10–100 µL (Fe^2+^ chelating effect)	The lowest peroxide value with respect to oleoresins, synthetic antioxidants and essential oil from dry rhizomes. More efficient inhibitory effect of malondialdehyde Higher DPPH radical scavenging, as well as Fe^2+^ chelating abilities than the dry ones (21.4% ar-turmerone, 7.2% α-santalene and 6.6% ar-curcumene) Higher DPPH radical scavenging activity than BHA and BHT	[164]
21.4% ar-Turmerone 7.2% α-Santalene 6.6% ar-Curcumene	100–200 Meq/kg (peroxide value) 0.04–0.08 TBA value 15–20 µL (DPPH) 10–100 µL (Fe^2+^ chelating effect)	Higher DPPH radical scavenging activity than BHA and BHT	[164]
20.4% α-Phellandrene 19.8% α-Turmerone 10.3% 1,8-Cineole 7.35% β-Turmerone	28.1 ± 1.45 mmol Trolox/L (PLC)	Free radical-scavenging potential twice higher than that of Trolox (~60 *vs.* 28.2%, respectively) Antioxidant activity (72.4%) near the values of the reference essential oil *Thymus vulgaris* (90.9%) and butylated hydroxyanisole (BHA) (86.74%)	[217]
22.8% β-Sesquiphellandrene 9.5% Terpinolene	IC_50_ 3.227 mg/mL (DPPH) IC_50_ 1.541 mg/mL (ABTS) 1 mg/mL (antiperoxidative)	Hydrogen donating properties and reducing power. Potential option to prevent oxidative deterioration of fat containing food products	[256]
35.46% Turmerone 20.61% Cumene 13.82% ar-Turmerone	30 µL/mL	Suppression of oxidase enzyme activity of the fresh-cut “Fuji” apples by 9% when incorporated in a starch/carboxymethyl cellulose edible coating	[261]
38.7% ar-Turmerone 14.2% α-Turmerone	10 µL	Negligible DPPH radical scavenging activity with respect to other different essential oils (cinnamon, clove, green tea, lemon eucalyptus, rosemary, oregano and its main compound carvacrol)	[262]

On the other hand, other *Curcuma* spp. essential oil with very different chemical compositions have also demonstrated strong and dose-dependent antioxidant abilities. In this sense, *C. zedoaria* (17.72% curzerenone, 15.85% γ-eudesmol acetate and 6.50% germacrone) and *C. angustifolia* (29.62% epicurzerenone, 10.79% curzerenone and 6.12% *trans*-β-terpineol) rhizome essential oils showed higher DPPH (IC_50_ 2.58 ± 077 µg/mL and 12.53 ± 0.14 µg/mL) and ABTS (IC_50_ 1.28 ± 0.05 µg/mL and 5.53 ± 0.29 µg/mL) radical scavenging ability, as well as reducing power (EC_50_ 4.77 ± 0.14 µg/mL and 5.68 ± 0.11 µg/mL) than BHT and ascorbic acid (DPPH: 19.07 ± 0.17 and 5.31 ± 0.2 µg/mL, ABTS: 14.19 ± 0.21 and 1.51 ± 0.32 µg/mL and reducing power: 9.61 ± 0.18 and 5.21 ± 0.13 µg/mL, respectively) [194]. The leaf essential oil of *C. angustifolia* (33.2% curzerenone, 18.6% 14-hydroxy-δ-cadinene and 7.3% γ-eudesmol acetate) showed even higher DPPH and ABTS free-radical scavenging (4.06 ± 0.06 and 1.35 ± 0.14 µg/mL, respectively), as well as reducing (EC_50_ 2.62 ± 0.25 µg/mL) activities, than the rhizome oil and the standard references [128]; *C. amada* rhizome oil (40% β-myrcene, 11.78% β-pinene and 10% ar-curcumene) and the essential oil obtained from the pulverized rhizome of *C. petiolata* (83.99% 2-methyl-5-pentanol) presented moderate antioxidant activity in comparison to the extracts and standard references [133,263].

*C. longa* rhizome essential oil has also exhibited strong antioxidant potential when combined with other essential oils—for instance, *Z. officinale*. In this case, the combination of both showed higher DPPH radical scavenging activity (IC_50_ 3.75 µL/mL vs. 4.28 and 7.19 µL/mL), as well as stronger β-carotene–linoleic acid bleaching (65.24% vs. 59.88 and 55.82%) than *C. longa* and *Z. officinale* oils alone, respectively [235]. This last test has been commonly used to compare the lipid peroxidation inhibitory activity of either individual compounds or mixtures, despite possible scattered results due to different factors like the chemical composition and extracting solvent [264].

Overall, the genus *Curcuma* and its derived products have been popularly used as food additives to confer special beneficial properties, which include colouring, preservation and healthy effects. Particularly, the biopreservative properties of *C. longa* rhizome oil can meet the needs of the agri-food industry. Its suitability as a natural alternative to synthetic antioxidants has been broadly corroborated through many in vitro and in vivo tests, obtaining interesting results replacing the reference synthetic antioxidants. So much so that this essential oil is being included in food coatings to keep them much longer. Moreover, further investigation regarding the most appropriate application of *C. longa* rhizome oil, as well as combinations with other, different essential oils, is being carried out, with the aim of trying to enhance its antioxidant potential and being finally implemented in the sustainable agri-food industry.

## 5. Conclusions

Consumers demand natural, safer and greener products, as well as sustainable food technologies, from the agri-food industry. However, an equilibrium between meeting consumer expectations and achieving the maximum efficiency in industrial production according to Green Chemistry is required.

The potential applications of numerous plant products in the agri-food industry have been widely investigated. Amongst them, the essential oil extracted from the rhizome of *C. longa* (species popularly known for its medicinal and culinary benefits) has demonstrated a high antimicrobial potential against a broad spectrum of plagues in crops and food-spoilage microorganisms, as well as significant phytotoxic effects against diverse weeds that are considered truly a threat for agricultural production and ecology. Besides, it has exhibited interesting antioxidant activity that would avoid postharvest decay and extend food shelf-lives.

This versatility is mainly due to the characteristic chemical composition of *C. longa* rhizome essential oil. Usually, sesquiterpenes constitute the main phytochemical group identified, and turmerones are the most representative components. However, this pattern is subjected to changes depending on countless internal (genetics) and external (geographic location, cultivation conditions, post-harvest processing, etc.) factors. For this reason, predictive models need to be developed to previse the chemical composition of *C. longa* rhizome essential oil according to the conditions surrounding the plant. In this way, the control of these factors is useful to obtain a high-yield essential oil with the aimed chemical composition, convenient for carrying out a specific activity in the agri-food industry in an optimum way.

Given the nature of these products (complex mixtures of volatile compounds), one of the first processes to take into account is the extraction technique chosen. Despite that the conventional methods (steam distillation, hydrodistillation, etc.) are still the most commonly used, there is a current tendency to employ the novel ones (SFE, SWE, SFME, MAE, etc.) that offer several advantages, such as the reduction of costs, of extraction times, energy consumption, etc., in an attempt to offer higher-quality *C. longa* rhizome essential oil in the lowest time possible and with the minimum residues produced. For its total implementation, further research is needed to achieve the most efficient extraction that allows obtaining a chemical composition enriched in the active component to elucidate its mechanisms of action, encapsulating techniques of *C. longa* essential oil for its preservation and/or release against external conditions (temperature, oxygen, etc.), as well as to determine the threshold application with which it would neither damage crops nor affect the organoleptic properties of food products, are necessary research prior to their employment in the agri-food industry.

The sustainable and efficient encapsulation of *C. longa* rhizome oil represents the ultimate step for its implementation in the agri-food industry. Current research is oriented to solve the limitations when applying turmeric essential oil (volatility, instability under certain conditions and hydrophobicity), with the aim of longer preserving its numerous benefits and improving its performance. Biodegradable and biocompatible products as edible alginate-based films with turmeric represent advantages over traditional plastic containers, increasing the antioxidant capacity and extending the shelf-lives of the final products. Many encapsulation methods, including β-cyclodextrines, chitosan–alginate, microemulsions, nanoparticles etc., have been described to enhance the curcumin bioavailability. They represent potential options to also enhance the beneficial properties of the essential oil of *C. longa* rhizome and its components, as well as controlling their release. A complex study regarding the cost-efficiency and sustainability, as well as threshold concentrations not to harm crops and food, have to be taken into account.

## Data Availability

No new data were created or analysed in this study. Data is not applicable to this article.
